# Aquaporins in Salivary Glands: From Basic Research to Clinical Applications

**DOI:** 10.3390/ijms17020166

**Published:** 2016-01-27

**Authors:** Christine Delporte, Angélic Bryla, Jason Perret

**Affiliations:** Laboratory of Pathophysiological and Nutritional Biochemistry, Faculty of Medicine, Université Libre de Bruxelles, 808 Route de Lennik, Blg G/E CP 611, Brussels B-1070, Belgium; angelic.bryla@ulb.ac.be (A.B.); jason.perret@ulb.ac.be (J.P.)

**Keywords:** aquaporin, expression, saliva secretion, salivary gland, subcellular localization, pathophysiology, physiology

## Abstract

Salivary glands are involved in saliva secretion that ensures proper oral health. Aquaporins are expressed in salivary glands and play a major role in saliva secretion. This review will provide an overview of the salivary gland morphology and physiology of saliva secretion, and focus on the expression, subcellular localization and role of aquaporins under physiological and pathophysiological conditions, as well as clinical applications involving aquaporins. This review is highlighting expression and localization of aquaporins in human, rat and mouse, the most studied species and is pointing out possible difference between major salivary glands, *i.e.*, parotid, submandibular and sublingual glands.

## 1. Introduction

Aquaporins (AQPs) are a family of transmembrane protein channels accounting for transcellular water permeability [1,2]. In addition to being permeable to water, some AQPs can be permeable to small solutes, including cations and glycerol, and gases [1,2]. Based on their structure and permeability characteristics, AQPs are subdivided into classical AQPs, primarily permeable to water but also to ions and gases (AQP0, AQP1, AQP2, AQP4, AQP5, AQP6, AQP8) [1,3,4]; aquaglyceroporins permeable to glycerol and other solutes in addition to water (AQP3, AQP7, AQP9, AQP10) [1,5,6]; and non-classical AQPs of uncertain permeability to water and/or glycerol (AQP11, AQP12) [7].

The expression and function of AQPs has been studied in salivary gland where they contribute to saliva secretion. This review will provide an overview of the salivary gland morphology and physiology of saliva secretion and focus on the expression, subcellular localization and role of aquaporins under physiological and pathophysiological conditions, as well as clinical applications involving aquaporins.

## 2. Morphology of Salivary Glands

### 2.1. Epithelial Cell Types

Salivary glands, surrounded by a capsule, are composed of many divisions called lobes which are further subdivided into smaller sections called lobules. Each lobule is separated by an extensive septum made of connective tissue.

**Figure 1:**
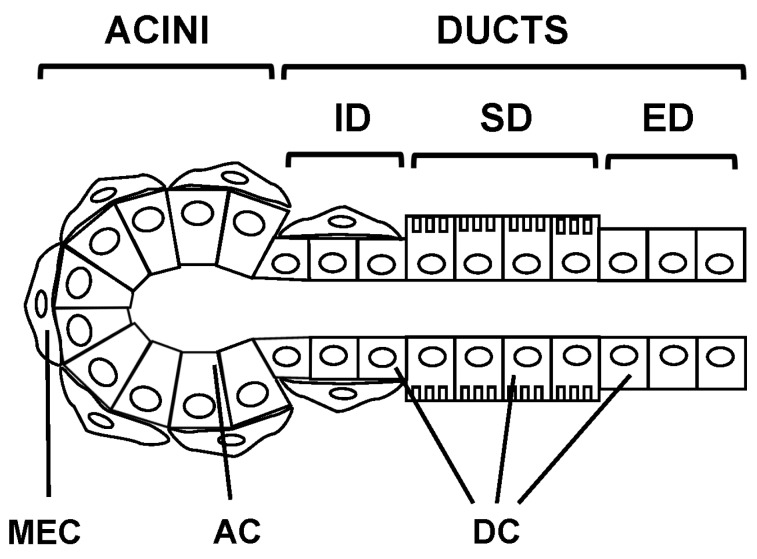
Morphology of salivary glands. Salivary glands are made of three epithelial cell types: acinar (AC), ductal (DC) and myoepithelial cells (MEC). Acinar cells organized into acini connected to a network of ducts classified into intercalated (ID), striated (SD) and excretory ducts (ED).

Salivary glands are made of three epithelial cells types: acinar, ductal and myoepithelial [8,9,10,11] (Figure 1). The acinar cells form acini structures responsible for fluid secretion draining into the lumen of the ducts consisting of ductal cells [8,9,10,11].

The secretory portion of salivary glands can be classified into mucous, serous and seromucous acini. Following hematoxylin-eosin staining, these three types of acini can be easily distinguished based on their morphological and staining characteristics. Indeed, mucous acini consist of large columnar acinar cells with intra flattened nuclei located close to the basal membrane. Serous acini contain smaller basophilic pyramidal cells with spherical nuclei. Seromucous acini have large mucous portion with a crescent-shaped serous portion called serous demilune.

The acini-secreted fluid drains to the mouth through an extensive network of ducts classified as intralobular, interlobular and interlobar. Intralobular ducts are localized within the lobules of the salivary glands and will empty into interlobular ducts. Two types of intralobular ducts can be distinguished: the intercalated ducts and the striated ducts. Intercalated ducts, connecting the acini to the striated ducts, consist of simple short cuboidal cells resting on a basement membrane. Striated ducts, connecting the intercalated ducts to the interlobular ducts, consist of simple columnar cells displaying longitudinal striations due to mitochondria at the base of the cells. Interlobular ducts, connecting striated ducts to interlobar ducts, consist of simple columnar cells and are located between the lobules of the salivary glands. Interlobar ducts—also called excretory ducts—connecting interlobular ducts to the oral cavity, are very large ducts located between the lobes (Figure 1). Myoepithelial cells are present in most salivary glands. They usually associate with both serous acini and serous demilune portion from seromucous acini, as well as mainly intercalated ducts, and to a lesser extent to interlobar ducts and interlobular ducts (Figure 1). Myoepithelial cells surrounding acini are stellate in shape, while those associated with ducts are elongated and oriented parallel to the ductal axis.

### 2.2. Salivary Glands Types

Salivary glands can be subdivided into three major salivary glands—parotid, submandibular, sublingual—and numerous minor salivary glands—forming small packages located in the labial, palatine, buccal, lingual and sublingual submucosae [8,9,10,11]. Each major salivary gland drains saliva to the mouth cavity by a single interlobar duct, while minor salivary glands use multiple interlobar ducts.

Human and rodent parotid glands are exclusively composed of serous acini. Human submandibular gland is composed of both serous, mucous and seromucous acini, while the rodent has only serous acini. Human submandibular glands contain more serous acini than mucous and serous acini. Human and rodent sublingual glands consist of centrally-located mucous acini and peripherally-located seromucous acini. Most human and rodent minor salivary glands are composed of mucous and seromucous acini [12].

## 3. Expression and Localization of AQPs in Salivary Glands

### 3.1. Human

In all human salivary glands, AQP1 expression was localized to myoepithelial cells [13] and endothelial cells [14,15,16]. AQP3 expression was detected in all salivary glands at basolateral membranes of both serous and mucous acini, but not to ducts [15,16,17]. Despite the presence of AQP4 mRNA in all salivary glands, AQP4 protein has not been confirmed [15,16]. AQP5 expression has exclusively been localized to the apical membrane of serous acini [15,16,18]. Both AQP6 and AQP7 mRNAs, but not the proteins, have been detected to submandibular glands [16]. Table 1 summarizes the expression and localization of AQPs in salivary glands from human, but also from rat and mouse (see Section 3.2. and Section 3.3).

### 3.2. Rat

In rat submandibular glands, the expression of AQP1, AQP3 and AQP5 has been detected during both pre- and post-natal development [19]. The expression of AQP1 was confined to endothelial cells [19,20,21,22]. The expression of AQP4 protein in adult rat submandibular glands remains controversial [23,24,25]. AQP5 expression was localized to the apical membranes of acini [19,26], but remains controversial in the ducts [19,24,27,28].

Both AQP1 and AQP5 mRNAs and proteins have been detected in endothelial cells and acini, respectively, from adult rat parotid glands [20,25,29,30]. AQP6 expression has been localized to plasma membrane and secretory granule membrane [31]. AQP8 expression was located to myoepithelial cells [32,33,34].

### 3.3. Mouse

Mouse salivary glands express AQP1 in endothelial and myoepithelial cells [35]. AQP3, AQP4 and AQP8 are expressed at the basolateral membrane of acinar cells and in ductal cells [35]. In mouse submanbibular, parotid and sublingual glands, acinar cells express AQP5 [36,37,38]. In mouse submandibular and sublingual glands, endothelial cells express AQP7 and satellite glial cells of parasympathetic ganglia express AQP4 [38]. While both AQP9 mRNA and protein have been detected in both mouse submandibular and sublingual glands [36,38,39], the precise cell distribution of AQP9 remains to be assessed. AQP11 is expressed in ductal cells [36,38].

**Table 1:** Expression and localization of AQPs in salivary glands.

AQP	Human	Rat	Mouse
AQP1	EC, MEC	EC	EC, MEC
AQP3	AC (BM)	AC	AC (BM), DC
AQP4	N.D. (mRNA)	AC (controversial)	AC (BM), DC
AQP5	AC (AM)	AC, DC (controversial)	AC
AQP6	N.D. (mRNA)	AC	N.D.
AQP7	N.D. (mRNA)	N.D.	EC.
AQP8	N.D.	MEC	AC (BM), DC
AQP9	N.D.	N.D.	N.D. (mRNA)
AQP11	N.D.	N.D.	DC

AC: acinar cells; AM: apical membrane; BM: basolateral membrane; DC: ductal cell; EC: endothelial cell; MEC: myoepithelial cell; mRNA: the transcript has been detected but not the protein; N.D.: not determined. AQP2, AQP10 and AQP12 have not been detected in any of the salivary glands from the above-mentioned species and AQP10 is a pseudogene in mouse.

## 4. Physiology of Saliva Secretion

Humans secrete 750 to 1000 mL of saliva per day. Mainly submandibular and minor salivary glands account for basal saliva secretion, while parotid glands account for stimulated saliva secretion. Mucous acini secrete thick and viscous fluid rich in mucins, while serous acini secrete non-viscous proteinaceous fluid rich in amylase and lysozyme. Saliva is composed of water (by far the major component), ions and proteins (among which proline-rich proteins, mucins, amylases, cystatins, histatins and statherin represent the major protein families) [40]. Saliva ensures mucosa and teeth lubrication and antimicrobial defenses, taste, digestion, bolus formation, maintenance of mucosa, food clearance, teeth mineralization and buffering [40]. Therefore, saliva secretion dysfunction leads to multiple clinical manifestations [41].

**Figure 2:**
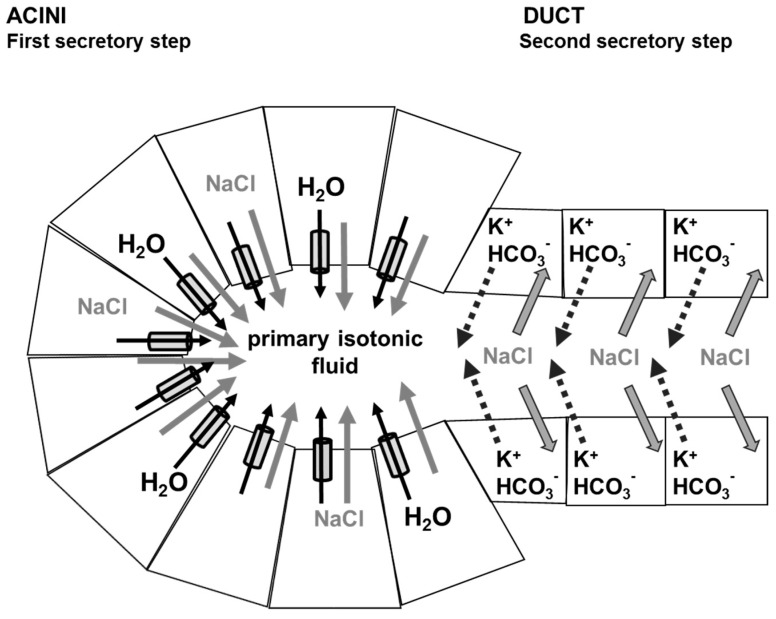
Physiology of saliva secretion and role of AQP5. During the first secretory step, acinar cells secrete NaCl which creates a transepithelial osmotic gradient driving consequently water flow through AQP5 and paracellular pathway, leading to a primary isotonic fluid secretion. During the second secretory step, ductal cells, impermeable to water, reabsorb most NaCl and secrete K^+^ and HCO_3_^−^. This leads to the formation a final hypotonic saliva flowing to the mouth cavity.

Current salivary fluid secretion model involves two distinct steps (Figure 2). During the first step, an isotonic-like fluid rich in NaCl is secreted by acinar cells involving several ionic transporters [42,43]. Sequentially, the “primum movens” to water movement is thought to be the accumulation of NaCl in acini lumen (due to the action of several ionic transporters). The accumulation of NaCl generates a transepithelial osmotic gradient driving consequently important transcellular [42,43], and possibly paracellular [44], water flux (Figure 2). The resulting primary fluid secretion reach the ductal lumen and undergoes modifications of its composition. Indeed, during the second step, the ductal cells, which are relatively impermeable to water [45], reabsorb most of the Na^+^ and Cl^−^ and secrete HCO_3_^−^ and K^+^ (Figure 2). This leads to a modification of the primary fluid composition, generating a final hypotonic saliva [42,43].

Under resting conditions salivary glands maintain a basal saliva secretion, while upon demand, upregulation of saliva secretion is achieved by autonomic parasympathetic and sympathetic nerve stimulations [42,43,46]. Parasympathetic nerve stimulation is mainly mediated by acetylcholine. Acetylcholine induces most of saliva fluid secretion, by activating mainly M3 and to a lesser extent M1 receptors which activate intracellular signaling events leading to calcium mobilization [46,47,48]. Sympathetic nerve stimulation is mainly mediated by noradrenalin. Noradrenalin induces essentially enzyme secretion by activating β-adrenergic receptors which activate intracellular signaling cascades leading to intracellular cAMP increase [46,47,49]. In addition, non-cholinergic non-adrenergic (NANC) stimulation—involving vasoactive intestinal peptide (VIP), pituitary adenylyl cyclase activating polypeptide (PACAP) and ATP—has been implicated in protein and fluid salivary secretion, as well as in modulating both cholinergic and adrenergic nerve stimulations [50,51,52,53,54].

## 5. Role of Aquaporins in Saliva Secretion

The major role played by AQP5 in saliva secretion has been revealed using AQP5 knockout mice [49,55,56]. Indeed, as compared to wild type mice, AQP5 knockout mice displayed a 60% decrease in pilocarpine-stimulated saliva secretion, as well as a more viscous and hypertonic saliva [55,56]. In addition, water permeability decreased by 65% and 77% in parotid and sublingual acinar cells submitted to osmotic challenges [56]. Knockout mice for AQP1, AQP4 and AQP8 revealed that these AQPs are unlikely involved in saliva secretion as their pilocarpine-stimulated saliva productions were comparable to that of wild type mice [55,57,58]. Moreover, the involvement of other AQPs in saliva secretion has not been demonstrated. In the current saliva secretion model, transcellular water flux occurring during the first step of saliva secretion is most likely ensured by AQP5 [42,43]. An osmosensor feedback model suggests that an osmosensor, most likely AQP5, controls the tonicity of the transported fluid by mixing transcellular and paracellular water flows [59]. Opposing, a saliva secretion model based on transcellular-only osmotic mechanism predicts the results obtained using AQP5 knockout mice [60,61]. More recently, a multiscale modeling of saliva secretion was constructed based on the assumption of osmotically driven water flow across acinar cells, among several other assumptions [62]. Therefore, the precise contribution of both transcellular (likely mediated by AQP5) and paracellular water flows to saliva secretion still remain unclear.

In acinar cells, AQP5 traffics from intracellular vesicles to plasma membrane in response to acetylcholine [26,49,63,64]. In rat parotid glands, AQP5 also participates to the osmoregulation of acinar secretory granules [65].

## 6. Aquaporins and Pathophysiological Conditions

### 6.1. Sjögren’s Syndrome

Sjögren’s syndrome (SS) is a chronic autoimmune disease affecting predominantly women with respect to men (9:1 ratio), and characterized by lymphocytic infiltration of exocrine glands, mainly salivary and lachrymal glands [66]. SS is classified as either primary, when occurring alone, or secondary, when occurring along with another autoimmune diseases such as rheumatoid arthritis, systemic lupus erythematosus or myositis. Although the origin of the disease remains unknown, epithelial cell activation is considered to play a central role in the multifactorial pathogenesis of SS [67,68,69,70]. Several autoantibodies have been detected in patients suffering from SS and associated with clinical features of SS; including against ribonucleoproteins (Ro/SS-A) and La/SS-B), CA19-9, muscarinic M3 receptors and pancreatic ductal cells [71,72,73,74].

In patients suffering from Sjögren’s syndrome, an abnormal localization of AQP5, with a predominant basolateral membrane localization instead of an apical membrane localization, and/or an altered AQP5 expression has been observed [18,75,76]. There are also data reporting no such alteration in AQP5 expression and distribution in patients suffering from Sjögren’s syndrome [77,78,79]. However, abnormal localization of AQP5 has been detected in several animal models for Sjögren’s syndrome including non-obese diabetic (NOD) mice [80,81,82], IQI/JIC mice [82], specific T-cell class IA phosphoinositide 3-kinase (r1∆T/r2n) knockout mice [82], mice immunized with submandibular gland autoantigen [83], and NOD/SCID.E2f1^−/−^ mice [84]. In NOD, IQI/JIC and r1∆T/r2n mice, AQP5 altered distribution appears to be linked to the presence of inflammatory infiltrates and acinar destruction [82]. Cytokines and autoantibodies directed against muscarinic M3 receptors may play a role in the altered distribution and/or expression of AQP5 observed in salivary glands from patients suffering from Sjögren’s syndrome and from animal models of Sjögren’s syndrome. Indeed, toll-like receptor activation and TNFα decrease AQP5 expression in salivary glands [85,86], while IFN-α (improving xerostomia in patients with Sjögren’s syndrome; [87,88] increase AQP5 expression [89]. In addition, autoantibodies directed against muscarinic M3 receptors inhibit AQP5 trafficking in salivary glands [90,91]. Furthermore, Rituximab (an anti-CD20 monoclonal antibody), which improves xerostomia in patients suffering from Sjögren’s syndrome and induces complete remission of lymphoma in some cases [92,93], increased AQP5 expression at the apical membrane of salivary gland acinar cells [93]. Altered AQP5 expression and/or localization could thereby participate to the pathogenesis of Sjögren’s syndrome, even though it could not totally account for saliva impairment [81].

A decrease in AQP1 expression has been documented in labial salivary glands biopsies from patients suffering from Sjögren’s syndrome, while no change has been detected in endothelial cells [17]. Rituximab increased AQP1 expression in myoepithelial cells and saliva flow in patients suffering from Sjögren’s syndrome [94]. AQP1 might be involved in saliva secretion as acetylcholine induced myoepithelial cells contraction and AQP1 trafficking [17]. However, data obtained from AQP1 knockout mice do not support this hypothesis [55,56]. Further studies are necessary to better understand the role of both AQP5 and AQP1 in xerostomia resulting from Sjögren’s syndrome.

### 6.2. Radiation Therapy

Most patients diagnosed with head and neck cancer (±500,000 new cases per year worldwide) will receive ionizing radiation therapy combined with surgery for their treatment [95]. However, salivary gland acinar cells lying in the radiation field will suffer substantial damage, with xerostomia as the ultimate outcome for the patients [96]. With respect to AQP5 expression, decrease/loss [97,98], as well as defect in AQP5 trafficking [99] have been observed in salivary glands from irradiated rats.

### 6.3. Diabetes

In 2030, diabetes prevalence is estimated to be 4.4% and to affect 366 million people worldwide [100]. Diabetes also represents a common cause of xerostomia [101,102]. Reduced saliva flow without altered AQP5 localization was observed in mice and rats with streptozotocin-induced type-1 diabetes [103,104]. Additional studies are required to fully appreciate the role of AQP5 in diabetic xerostomia.

### 6.4. Senescence

As age increases, salivation declines gradually in humans and mice [105]. During senescence, decreased AQP5 expression and/or trafficking might account partly for xerostomia [106].

## 7. Aquaporins and Clinical Applications

### 7.1. Medications

Cevimeline could represent a useful drug for xerostomia treatment, especially in diabetic patients and elderly individual. Indeed, in rats with streptozotocin-induced type-1 diabetes, as well as in senescent rats, cevimeline injection restored AQP5 trafficking [104,106,107]. Further studies must be performed to evaluate the potential beneficial effects of cevimeline for xerostomia of various etiologies.

Identification of new drugs capable of increasing AQP5 expression could provide new perspectives for the treatment of xerostomia. Recently, DNA demethylation agents have been shown to restore saliva secretion in senescent animals [108]. For obvious safety issues the usefulness of such drugs need to be ruled out in patients. However the observation of DNA methylation may open new avenues of investigation of gene regulation linked to long term gene expression alterations linked to senescence. Nevertheless, search for new drugs capable of increasing AQP5 expression should be encouraged.

### 7.2. Gene Therapy

Gene therapy, allowing DNA delivery into patient’s cells to treat diseases, has been used to treat xerostomia resulting from cancer irradiation therapy. Following such cancer irradiation therapy, it was hypothesized that, based on the current understanding of salivary gland physiology, ductal cells could generate an osmotic gradient (extracellular > intracellular) allowing fluid secretion if a facilitated water permeability pathway was introduced using a recombinant adenoviral vector coding for human AQP1 (AdhAQP1) [29,109]. AdhAQP1 was indeed shown capable to drive both AQP1 expression and osmotically-driven fluid secretion in epithelial cells [29,110,111]. In irradiated rat submandibular glands suffering from xerostomia, AdhAQP1 restored saliva secretion [29]. Similar data were obtained in miniature pigs and non-human primates [112,113,114]. In a subset of subjects with prior irradiated parotid glands, AdhAQP1 was shown to be safe in clinical trials, to induce increased parotid saliva flow and to relieve symptoms [115]. This clinical trial was the first to conduct gene therapy in the oral cavity for non-malignant condition and to use an AQP as a therapeutic agent. Persistence of hAQP1 expression in salivary gland cells from this subset of patients was likely associated with a lack of methylation of the hCMV promoter present in the adenoviral construct [116]. However, as adenoviral gene transduction allows transient expression of the transgene, further studies are required to engineer new viral vectors that would allow more efficient and persistent expression of a transgene, such as for instance hAQP1, in salivary gland. Despite this consideration, gene therapy using viral vector coding for an AQP still represent a promising therapy for patients suffering from xerostomia subsequent to head and neck irradiation therapy as well as to Sjögren’s syndrome.

### 7.3. Stem/Progenitor Cells and Tissue Regeneration Therapy

Stem/progenitor cells have been identified in salivary glands from mice, rats and humans [117]. These cells reside in the ducts of salivary glands [117] and some have been shown to express AQP5 [118,119]. The number of stem/progenitor cells remaining in a damaged gland is hypothesized to determine the regenerative capacity of the gland [117]. However, it has been recently proposed that this hypothesis should be revised in light of the capacity of self-renewal of differentiated cells [120]. The use of transplanted stem/progenitor cells requires the long-term survival of the transplanted cells and a better understanding of the potential risks and shortcomings of this therapy. Despite these limitations, increasing amount of data suggest that stem/progenitor cells-based therapies could represent a promising strategy for the treatment of xerostomia resulting from salivary gland damage following head and neck radiation therapy or Sjögren’s syndrome [117,118,119].

## 8. Conclusions

Salivary glands are involved in saliva secretion. Several AQPs are expressed in salivary glands, including acinar and ductal cells involved in the saliva formation process. Among these AQPs, AQP5 plays a major role in saliva secretion. Altered expression, localization and/or trafficking of AQP5 have been identified in salivary glands following xerostomia of multiple origins. Several therapeutic strategies aiming at relieving xerostomia could arise from the development of drugs targeting AQP5. Gene therapy using AQP as a therapeutic agent, as well as tissue regeneration therapy using stem/progenitor cells represent promising therapies to treat xerostomia following head and neck cancer irradiation therapy or Sjögren’s syndrome.
